# Temporal Analysis of Patients Eligible for Randomized Controlled Trials in Total Hip Arthroplasty

**DOI:** 10.1016/j.artd.2026.101995

**Published:** 2026-05-25

**Authors:** Brynn P. Charron, Ethan Pieterman, Brent Charron, Elia Arbana, Brent A. Lanting

**Affiliations:** a Western University, Schulich School of Medicine & Dentistry, London, Ontario, Canada; b London Health Sciences Centre, London, Ontario, Canada; c Western University, London, Ontario, Canada; d University of Windsor, Windsor, Ontario, Canada

**Keywords:** Total hip arthroplasty, Methodology, Generalizability, Patient demographics, Exclusion criteria, Research design

## Abstract

**Background:**

There has been a shift in the demographics of patients undergoing elective lower extremity joint arthroplasties in recent years. This study aimed to assess whether research has kept pace with changing demographics through analyzing inclusion and exclusion criteria of randomized controlled trials (RCTs) relevant to total hip arthroplasties (THAs).

**Methods:**

Cross-sectional analysis of RCTs studying primary THAs indexed on ClinicalTrials.gov was performed. Trial characteristics regarding inclusion and exclusion criteria of 706 studies initiated between 1994-2023 were analyzed. Sample size, type of intervention, year of study initiation, length of study, and inclusion/exclusion criteria were evaluated.

**Results:**

An increasing number of THA-based RCTs were initiated over the study period (1994-2004: 5.18 studies/y, 2021-2023: 36.7 studies/y). The majority of included RCTs had an upper age exclusion criterion (1994-2004: 65.9%, 2021-2023: 53.6%). Body mass index (BMI) restrictions have become more common than previously (1994-2004: 5.3%, 2021-2023: 29.1%); however, the range has expanded to include higher BMI limits. The most common exclusion criteria based upon health comorbidities included psychiatric health and cognitive impairment (36.4%), neurologic and neuromuscular dysfunction (32.6%), and systemic infections (31.4%).

**Conclusions:**

Regarding elective primary THA, it is recognized that the age and BMI of patients have shifted substantially over recent decades. This study revealed that restrictions on participant age continue to be prevalent. Although the average BMI of patients undergoing elective THA is rising, BMI is increasingly being implemented as an exclusion criterion. Critical assessment of inclusion and exclusion criteria for all studies, especially RCTs, given their associated cost and potential impact, requires further attention in orthopaedics.

## Introduction

The incidence of total hip arthroplasty (THA) surgeries continues to steadily rise worldwide [1]. In 2020, 55,000 THAs were completed in Canada alone [2]. Life expectancy and conditions with well-researched associations to surgical outcomes have changed significantly over the past 3 decades. Global life expectancy has risen from 64 years old in 1996 to 73 years old in 2024, and obesity rate has risen from 27% to 43.5% over a similar period.3,4 As global populations age and the prevalence of conditions such as obesity and malignancy rise, the demographic profile of patients undergoing THAs is shifting to reflect these trends [3,5]. This changing landscape presents new challenges for clinical research, particularly in the selection and design process of randomized controlled trials (RCTs) for prospective data collection.

RCTs are considered the gold standard regarding clinical research design [4,6]. RCTs aim to minimize bias and ensure the internal validity of their findings by employing stringent inclusion and exclusion criteria. These criteria are designed to create homogenous study populations, thereby reducing variability and enhancing replicability. External validity, a factor that describes the generalizability of a study, is greatly reduced when the sample group does not closely relate to the general population [5,7]. For this reason, there is significant concern regarding the inclusion and exclusion criteria of RCTs and their potential to limit the generalizability of orthopaedic studies.

One area of concern that must be explored is whether the criteria used in THA-based RCTs have evolved to keep pace with the changing patient demographics. For example, an increasing number of patients younger and older the typical age range are undergoing THAs, prompting the question of whether age restrictions in trials are accurately reflecting these trends [[6], [7], [8], [9]]. Similarly, as obesity and comorbid conditions such as mental health diagnoses and malignancy become more prevalent, it is important to assess whether these factors are still frequently used as exclusion criteria [[8], [9], [10], [11], [12]].

The objective of this study is to analyze temporal trends in the inclusion and exclusion criteria of RCTs focused on THAs. Specifically, this study examines how patient demographic and health comorbidities are addressed in RCT criteria over the past 3 decades. By understanding these trends, the study aims to provide insight into the evolving landscape of clinical research and associated implications on RCT generalizability.

## Material and methods

A cross-sectional analysis of RCTs studying elective primary hip arthroplasty procedures indexed on the ClinicalTrials.gov site was performed. As a publicly available data source that contains only summary data instead of individual patient data, the use of data from ClinicalTrials.gov did not require institutional ethics review board approval. ClinicalTrials.gov is a free database updated daily by investigators and clinicians setting up clinical trials. The study followed the “Strengthening the Reporting of Observational Studies in Epidemiology” reporting guidelines.

**Figure 1:**
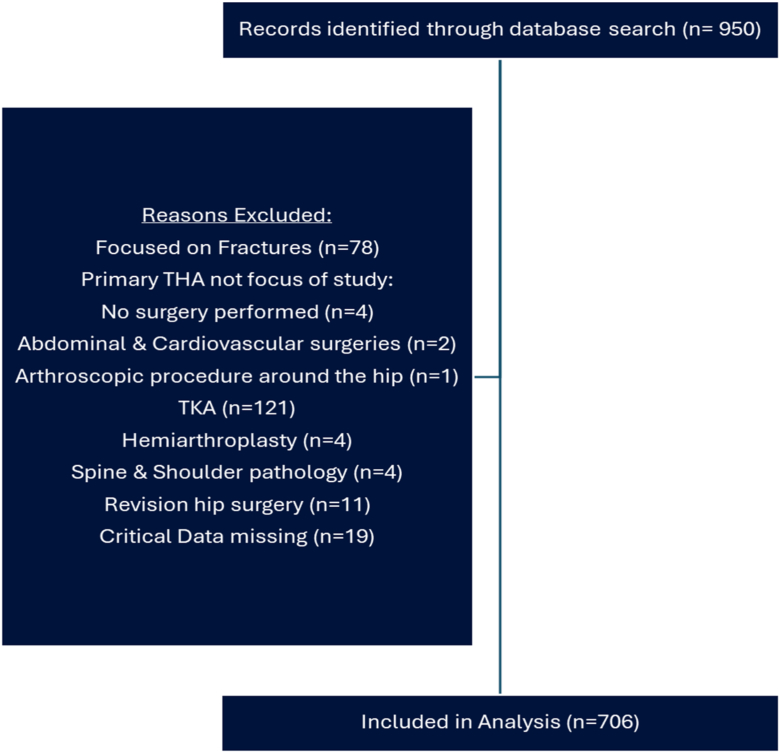
Flow diagram of indexed RCTs in Clinicaltrials.gov pertaining to THAs with reasons for exclusion from data analysis.

A search of the registered RCTs in the ClinicalTrials.gov database maintained by the National Library of Medicine was conducted on May 31, 2023. Keywords searched in the database were *hip*, *hip replacement*, and *hip arthroplasty*, with interventional studies being selected. Figure 1 depicts the flow of studies to the point of inclusion in analysis as well as reasons for exclusion. Two independent researchers (B.P.C. and E.P.) screened studies to ensure they were relevant to primary THAs. Information from the 706 studies retained in the analysis was extracted, including sample size, type of intervention, timing of intervention in relation to surgery, year of study initiation, length of study, inclusion and exclusion criteria. Studies initiated between January 1, 1994, and May 31, 2023, were analyzed. Data were verified through consensus. Frequencies were calculated for each type of exclusion criteria, stratified by study start date in 3-year intervals. The Fisher exact test was used to examine changes in frequencies over time. *P* values were considered significant at *P* < .05 using a Hochberg step-down procedure and were 2-sided. Regression modeling was utilized and coefficient of determination was reported. Data analysis included descriptive statistics utilizing Microsoft Excel (Microsoft Excel 2021).

## Results

**Figure 2:**
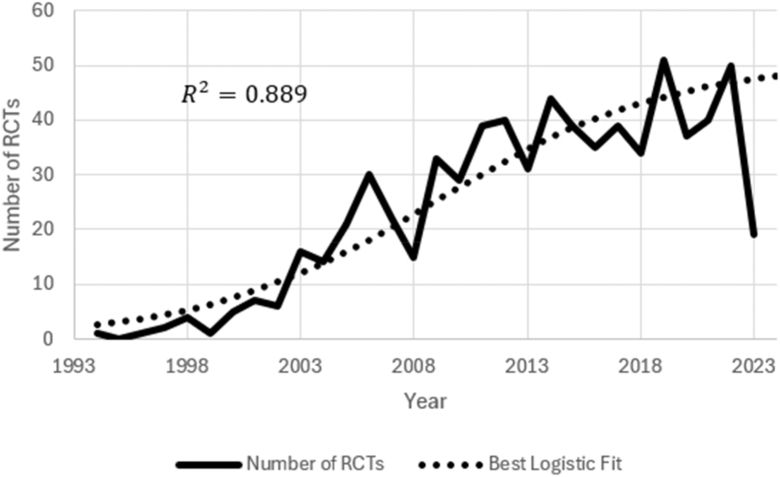
Number of THA RCTs per year from 1994 to 2022.

The analysis included 706 RCTs focused on primary THAs. From 1994 to 2022, the number of indexed trials drastically increased over time, as shown in Figure 2. Data regarding the number of THA RCTs conducted in 2023 were excluded given that the search was performed prior to the completion of the 2023 calendar year.

**Figure 3:**
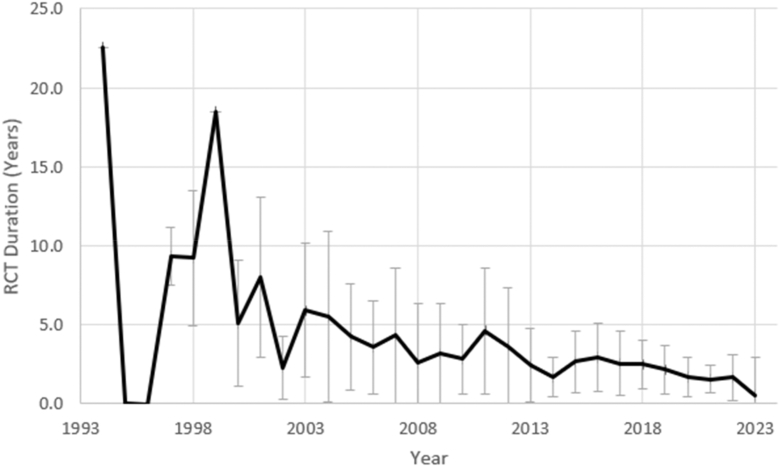
Average duration of THA RCTs per year from 1994 to 2024.

Over the period assessed, the average study duration fell from 22.6 years in 1994 to 1 year in 2024, as shown in Figure 3. While this trend is exaggerated by a high prevalence of outliers in early years without many RCTs, exclusion of these anomalies still maintain a noticeable downward trend.

**Figure 4:**
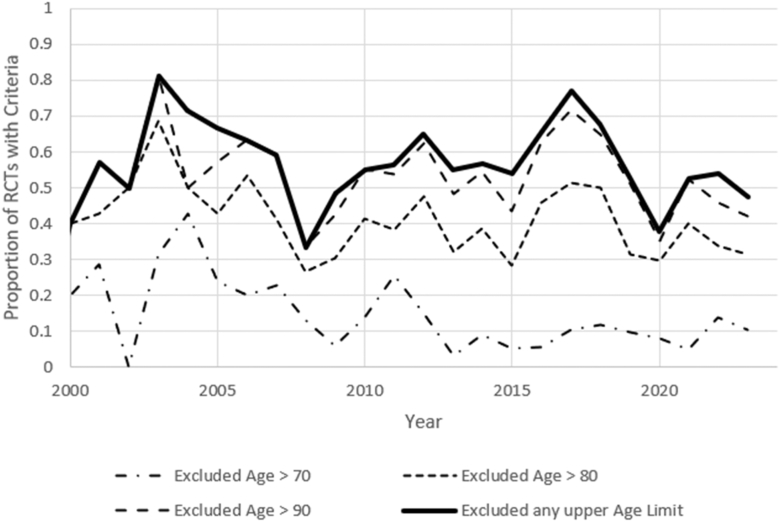
Proportion of THA RCTs implementing upper age exclusion criteria from 2000 to 2023.

Age exclusion criteria evolved over time, as shown in Figure 4. Lower age limits have remained the most common method of excluding participants based on age throughout the examined period. While the total proportion of studies implementing an upper age limit have remained relatively consistent, the proportion of studies excluding patients aged 80 years and above has decreased over time, indicating a general broadening of inclusion for older populations.

Upper limit body mass index (BMI) exclusion criteria have arisen over the past 30 years, with minimal to no RCTs implementing them at the start of the review period. Limits excluding participants with BMIs in excess of 40 kg/m^2^ in particular were more widely adopted.

Table 1 illustrates the characteristics of the included RCTs for the studied time period. Surgical procedures and implants (55.6%) and pharmaceutical therapies (28.5%) proved to be the most researched interventions. Studies examining intraoperative interventions (51.7%) were more common than postoperative (32.9%) and preoperative (15.4%) interventions. No significant changes in intervention type proportions with respect to time were noted. Table 2 outlines key exclusion and inclusion criteria frequency among the included RCTs. It should be noted that due to the limited number of RCTs conducted between 1994 and 2000, the time interval was expanded to encompass 8 years of data to allow for statistical analysis. Additionally, the final time interval spanning 2021-2023 contained fewer years than the other intervals. Therefore, when analyzing studies utilizing certain exclusion criteria, emphasis should be placed on percentages rather than the absolute number of studies. Analysis of inclusion and exclusion criterion demonstrated that a majority of included RCTs contained an upper age limit (1994-2004: 65.9%, 2021-2023: 53.6%). Furthermore, restrictions based on BMI increased in the more recent years compared to the past (1994-2004: 5.3%, 2021-2023: 29.1%). Regarding health comorbidities, the most common exclusion criteria included mental health and cognitive impairment (36.4%), neurologic and neuromuscular dysfunction (32.6%), and systemic infections (31.4%). Language fluency was utilized as a criterion in 14.5% of included RCTs (1994-2004: 7.33%, 2021-2023: 12.7%).

**Table 1:** Characteristics of RCTs on primary THAs registered in ClinicalTrials.gov.

Characteristic	Trials^a^ (N = 706)
Patients enrolled, median, patients	209
Length of study, median, years	2.92
Intervention type	
Procedure and implant design	392 (55.5)
Pharmaceutical	204 (28.9)
Other	69 (9.77)
Behavioral	24 (3.40)
Diagnostic	7 (0.99)
Dietary supplement	4 (0.57)
Radiation	4 (0.57)
Genetic	1 (0.14)
Combination	1 (0.14)
Relation to the surgery (number of RCTs)	
Intraoperative	365 (51.7)
Postoperative	232 (32.9)
Preoperative	109 (15.4)

a Data are present as number (percentage) of trials unless otherwise stated.

**Table 2:** Study inclusion and exclusion criteria in clinical trials on primary THAs registered in ClinicalTrials.gov.

Criteria	1994-2004 (N = 57)^a^	2005-2008 (N = 88)^a^	2009-2012 (N = 141)^a^	2013-2016 (N = 149)^a^	2017-2020 (N = 161)^a^	2021- 2023 (N = 110)^a^	Adjusted *P* value^b^
Excluded based on age							
<18 y	33 (58)	50 (57)	86 (61)	91 (61)	107 (66)	72 (65)	0.159
>70 y	28 (49)	38 (43)	71 (50)	82 (55)	86 (53)	50 (45)	0.078
>80 y	10 (18)	20 (23)	37 (26)	55 (37)	59 (37)	36 (33)	<0.001
>90 y	5 (9)	5 (6)	13 (9)	15 (10)	17 (11)	12 (11)	0.152
Any upper age limit	20 (35)	37 (42)	61 (43)	63 (42)	67 (42)	52 (47)	0.132
Excluded based on BMI							
>30 kg/m2	3 (5)	16 (18)	37 (26)	27 (18)	49 (30)	32 (29)	<0.001
>40 kg/m2	1 (2)	6 (7)	18 (13)	15 (10)	20 (12)	15 (14)	0.013
Any upper BMI limit	3 (5)	16 (18)	37 (26)	27 (18)	51 (32)	32 (29)	<0.001
Excluded based on a specific comorbidity							
Systemic infection	22 (39)	30 (34)	54 (38)	51 (34)	43 (27)	23 (21)	0.003
Cardiovascular disease	7 (12)	13 (15)	25 (18)	31 (21)	23 (14)	12 (11)	0.034
Lung	0 (0)	1 (1)	13 (9)	13 (9)	10 (6)	7 (6)	0.013
Renal	14 (25)	29 (33)	39 (28)	36 (24)	32 (20)	25 (23)	0.022
Psychiatric health and cognition	18 (32)	22 (25)	48 (34)	63 (42)	63 (39)	44 (40)	<0.001
Alcohol or drug misuse	20 (35)	33 (38)	44 (31)	42 (28)	47 (29)	34 (31)	0.136
Malignancy	18 (32)	16 (18)	37 (26)	25 (17)	16 (10)	11 (10)	<0.001
Diabetes	0 (0)	6 (7)	11 (8)	13 (9)	18 (11)	9 (8)	0.084
Neurologic or neuromuscular	17 (30)	21 (24)	42 (30)	54 (36)	56 (35)	39 (35)	0.048
Liver and gastrointestinal disease	7 (12)	12 (14)	25 (18)	28 (19)	23 (14)	20 (18)	0.266
Chronic pain diagnosis	1 (2)	8 (9)	8 (6)	9 (6)	14 (9)	10 (9)	0.070

a Data are present as number (percentage) of trials unless otherwise stated.

b *P* values were adjusted for multiple comparisons using a Hochberg step-down procedure.

## Discussion

This study highlights key trends in inclusion and exclusion criteria of RCTs focused on primary THAs over the past 3 decades. The findings indicate that while some criteria have evolved to better reflect the changing demographics of the patient population, others remain restrictive, potentially limiting the generalizability of research findings.

The number of THA RCTs registered each year increased throughout the reviewed period, as shown in Figure 2. Applying regression modeling to the number of THA RCTs over time reveals a logistic model was the best representation (R^2^ = 0.88). This indicates a higher rate of increase through 2003 to 2013 as compared to both before and after. Following the introduction of the concept of evidence-based medicine in the 1990s, this likely contributed to the boom of THA RCT registration in the 2000s (Evidence Based Medicine: What it is and what it is not).

The study duration as registered on clinicaltrials.gov was recorded from 1994 to 2023. The average study duration decreased over time substantially. Beginning in 2022, the average study duration was about 1 year. Given the significant increase in the number of RCTs registered annually, the shorter study duration is thought to reflect the relative funding limitations and productivity demands.

One of the most significant trends observed is the expansion of age-based inclusion criteria. The age range for eligible patients has widened, as evidenced in Figure 4. This reflects a more inclusive approach as the population undergoing THAs becomes more diverse [[6], [7], [8], [9]]. Specifically, as populations age worldwide, older adults are more likely to undergo THAs. As such, it is encouraging to see that clinical trials are adapting to be inclusive of this change. This shift ensures that results remain applicable to real-world clinical settings, further supporting the development of evidence-based practices.

**Figure 5:**
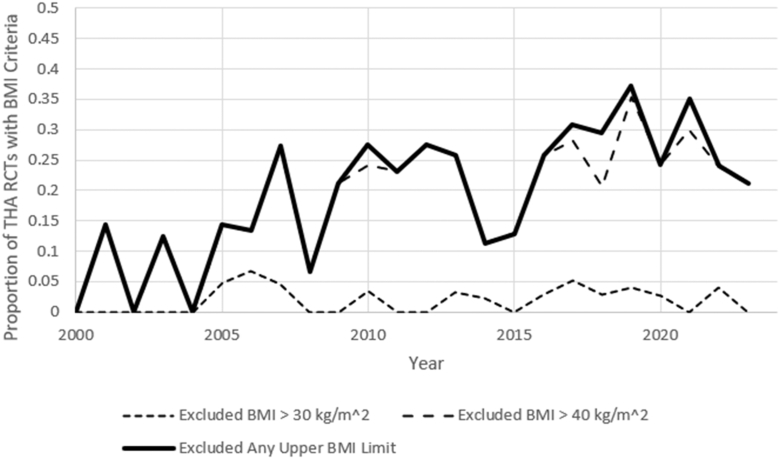
Proportion of THA RCTs implementing upper BMI exclusion criteria from 1994 to 2024.

The findings regarding BMI criteria are more complex. Research indicates that the BMI of the general population as well as that of individuals undergoing joint replacement surgery has risen over the past several decades. [8,10] The proportion of studies excluding participants based on an upper limit of BMI has increased over the examined period (Table 2, *P* < .001). However, the inclusion BMI range has also broadened to reflect the population. In studies that excluded participants based on BMI, earlier RCTs typically set exclusion criteria at a BMI of 30 to 40 kg/m^2^, while more recent studies raised their threshold to a BMI of greater than 40 kg/m^2^ (Fig. 5). This suggests a growing recognition of obesity as a significant factor in THA outcomes while also acknowledging the need for more inclusive research practices.

Comorbidities remained a significant exclusion criterion throughout the study period, with 85% of studies applying such restrictions. Systemic infections, neurological conditions, and psychiatric health disorders were consistently the most excluded conditions across all time periods. However, the specific exclusion criteria pertaining to regarding other excluded comorbidities, such as malignancy, systemic infection, and cognition, have evolved over time. The proportion of studies containing exclusion criteria based on the diagnosis of malignancy has significantly decreased since 2004 (pre-2004: 32%, 2021-2023: 10%; *P* < .001). It is recognized that over the past 3 decades, the diagnosis and survivorship of cancer has increased. In the United States, as of 2022, 48% of cancer survivors lived 10 years past their initial cancer diagnosis [[9], [10], [11], [12]]. The reduction of malignancy-based exclusion criteria may be a reflection of the increasing number of patients living long enough to undergo joint replacement surgery following a cancer diagnosis as well as the improved quality of life following cancer patients are experiencing. Additionally, the proportion of studies excluding patients based on systemic infection has decreased over time. This trend likely demonstrates the altered selection criteria for patients considered for THA. Previously, consideration for THA in patients with systemic infections may have been entertained. Currently, bacteremia is held as a contraindication to proceed with THA given what is known about periprosthetic joint infections as recently identified at the World Expert Meeting in Arthroplasty 2024 [11,13]. Exclusion based on psychiatric diagnoses and cognition significantly increased over the studied period. The exclusion criteria within the psychiatric diagnoses and cognition category referenced patients’ inability to provide informed consent, collaborate with the research team, or appropriately complete paperwork. This trend represents the increased focus on the ethical implications of research on patient subjects, and the push toward ensuring requirements set forth by research ethic committees are satisfied [12,14].

A limitation of this study is the nature of the ClinicalTrials.gov data collection process. Study inclusion and exclusion are listed at the time of registration; however, no data were available to ensure that adherence to these criteria was upheld or that investigators did not implement additional restrictions beyond those listed. ClinicalTrials.gov captures the majority of trials, however there is the potential for small or niche studies to go unregistered and be missed in the data collection process. Furthermore, explanations of exclusion criteria are not provided through ClinicalTrials.gov, which limits insight into researchers’ reasoning. Additionally, while this article assessed the change in inclusion and exclusion criteria of RCTs, the actual contents and results of the trials were not examined, which would provide additional perspective and insights on the evolution of the body of research over time. Future research should focus on investigating completed THA RCTs with respect to the adherence of inclusion and exclusion criteria, and the justification for these restrictions.

## Conclusions

The inclusion and exclusion criteria in RCTs for primary THAs have evolved over the past several decades to reflect key demographic changes in the patient population. Although the expansion of age criteria and broadening of BMI ranges reflect the changing landscape of the patient population, the large number of studies excluding patients based on health comorbidities raises concerns regarding the generalizability of research findings. To enhance the relevance of clinical research, it is crucial to strike a balance between maintaining the internal validity of studies and ensuring that findings are applicable to the diverse patient demographic. Future studies pertaining to joint replacement surgeries should prioritize inclusivity with respect to age and health comorbidities. By improving agreement between research and clinical patient populations, researchers and healthcare providers can ensure that research findings are relevant and applicable to the population at large.

## Ethics approval statement

Publicly available data were utilized and therefore ethics approval was waived.

## Informed consent statement

No individual patient data were utilized and therefore informed consent was not required.

## Funding

Institutional funding support from Western University was obtained.

## CRediT authorship contribution statement

**Brynn P. Charron:** Writing – original draft, Supervision, Project administration, Methodology, Investigation, Formal analysis, Data curation, Conceptualization. **Ethan Pieterman:** Investigation, Data curation. **Brent Charron:** Writing – review & editing, Validation, Methodology, Formal analysis, Data curation, Conceptualization. **Elia Arbana:** Writing – review & editing, Investigation, Formal analysis. **Brent A. Lanting:** Writing – review & editing, Supervision, Conceptualization.

## Conflicts of interest

B.A. Lanting is a paid consultant for Stryker, Smith & Nephew, and DePuy; receives research support from Smith & Nephew, DePuy, and Zimmer as a Principal Investigator; receives institutional support from DePuy, Smith & Nephew, Stryker, and Zimmer; all other authors declare no potential conflicts of interest.

For full disclosure statements refer to https://doi.org/10.1016/j.artd.2026.101995.

## Appendix A Supplementary data

Conflict of Interest Statement for Arbana

Conflict of Interest Statement for Charron

Conflict of Interest Statement for Pieterman

Conflict of Interest Statement for Charron

Conflict of Interest Statement for Lanting

## References

[1] Ruff G., Thomas J., Ashkenazi I., Grossman E., Davidovitch R., Schwarzkopf R. (2024). How has the total hip arthroplasty patient population changed? A ten-year analysis of total hip arthroplasty patients from 2013 to 2022: a retrospective, single-center Study. J Arthroplasty.

[2] Canadian Institute for Health Information (2023).

[3] World Health Organization Life expectancy at birth, 2026. Life expectancy at birth. https://www.who.int/data/gho/data/indicators/indicator-details/GHO/life-expectancy-at-birth-(years).

[4] World Health Organization Prevalence of overweight among adults, 2026. Overweight among adults, BMI >= 25, prevalence (age-standardized estimate) (%). https://www.who.int/news-room/fact-sheets/detail/obesity-and-overweight.

[5] Pirruccio K., Sloan M., Sheth N.P. (2019). Trends in obesity prevalence among total hip arthroplasty patients and the effect on surgical outcomes, 2008–2016. J Orthop.

[6] Gale R.P., Zhang M.J., Lazarus H.M. (2023). The role of randomized controlled trials, registries, observational databases in evaluating new interventions. Best Pract Res Clin Haematol.

[7] Paci M., Prestera C., Ferrarello F. (2020). Generalizability of results from randomized controlled trials in post-stroke physiotherapy. Physiother Can.

[8] Maman D., Fournier L., Steinfeld Y., Berkovich Y. (2024). Etiology, outcomes, and complications of total hip arthroplasty in younger patients: a nationwide big data analysis. J Clin Med.

[9] Antoniou J., Silotch C., Epure L., Antoniou A., Sampalis J. (2022). Elective total hip arthroplasties in Nonagenarians - age does matter: a national surgical quality improvement. Program Study.

[10] Williamson K., Nimegeer A., Lean M. (2020). Rising prevalence of BMI ≥ 40 kg/m2: a high-demand epidemic needing better documentation. Obes Rev.

[11] Tonorezos E., Devasia T., Mariottoa A.B., Mollica M.A., Gallicchio L., Green P. (2024). Prevalence of cancer survivors in the United States. J Natl Cancer Inst.

[12] Gallicchio L., Devasia T.P., Tonorezos E., Mollica M.A., Mariotto A. (2022). Estimation of the number of individuals living with metastatic cancer in the United States. J Natl Cancer Inst.

[13] Choe H., Indelli P., Kim T.-Y., Homma Y., Kigera J., Veloso Duran M. (2024). What are the absolute contraindications for elective total knee or hip arthroplasty?. J Arthroplasty.

[14] Humphreys K., Blodgett J.C., Roberts L.W. (2015). The exclusion of people with psychiatric disorders from medical research. J Psychiatr Res.

