# Supplementary material for: Temporal Analysis of Patients Eligible for Randomized Controlled Trials in Total Hip Arthroplasty
Source: Arthroplast Today. 2026 May 25;39:101995. doi: 10.1016/j.artd.2026.101995 (PMC13226844; doi:10.1016/j.artd.2026.101995)
Supplement: Conflict of Interest Statement for Lanting [file mmc5.docx]

# INDIVIDUAL CONFLICT OF INTEREST STATEMENT

***American Association of Hip and Knee Surgeons***

(Adopted from the American Academy of Orthopaedic Surgeons disclosure statement)

The following form **must be filled out completely and submitted by each author (example, 6 authors, 6 forms).**

**All items require a response. If there is no relevant disclosure for a given item, enter "*None*.”**

Manuscript Title: Analysis of Temporal Trends in Hip Arthroplasty Randomized Controlled Trials

1. Royalties from a company or supplier (The following conflicts were disclosed)

None

2. Speakers bureau/paid presentations for a company or supplier (The following conflicts were disclosed)

None

3A. Paid employee for a company or supplier (The following conflicts were disclosed)

None

3B. Paid consultant for a company or supplier (The following conflicts were disclosed)

Stryker, Smith and Nephew, DePuy

3C. Unpaid consultants for a company or supplier (The following conflicts were disclosed)

None

4. Stock or stock options in a company or supplier (The following conflicts were disclosed)

None

5. Research support from a company or supplier as a Principal Investigator (The following conflicts were disclosed)

Smith and Nephew, DePuy, Zimmer

6. Other financial or material support from a company or supplier (The following conflicts were disclosed)

Institutional support from DePuy, Smith and Nephew, Stryker, Zimmer

7. Royalties, financial or material support from publishers (The following conflicts were disclosed)

None

8. Medical/Orthopaedic publications editorial/governing board (The following conflicts were disclosed)

None

9. Board member/committee appointments for a society (The following conflicts were disclosed)

None

**Each author must sign AND print or type his/her name, date and submit a separate form**

In addition, one BLINDED Conflict of Interest form (no author names used) should be submitted per manuscript with all author disclosures.


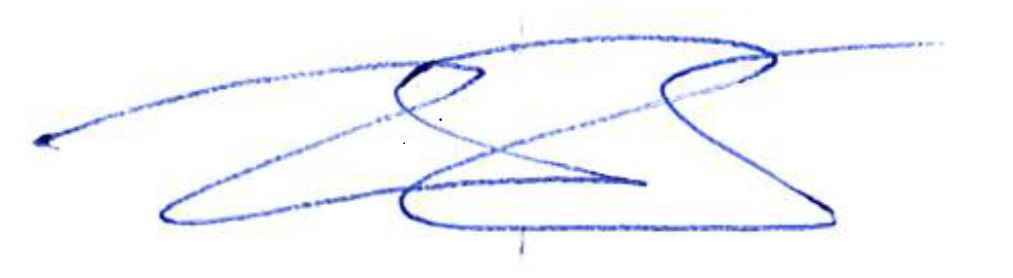


Brent Lanting November 16, 2024

Author Name (Print or Type) Author Signature Date
